# Bioelectrical impedance analysis in the BaSAlt cohort-study: the phase angle as an additional parameter for sarcopenia quantification among German nursing home residents?

**DOI:** 10.1007/s41999-023-00780-3

**Published:** 2023-04-13

**Authors:** Daniel Haigis, Silas Wagner, Ansgar Thiel, Andreas M. Nieß, the BaSAlt study team

**Affiliations:** 1 Department of Sports Medicine, University Hospital of Tuebingen, 72076 Tübingen, Germany; 2 Interfaculty Research Institute for Sport and Physical Activity, University of Tuebingen, 72074 Tübingen, Germany; 3 Institute of Sport Science, Faculty of Economics and Social Sciences, Eberhard Karls University of Tuebingen, 72074 Tübingen, Germany

**Keywords:** Bioelectrical impedance analysis, Muscle quality, Phase angle, Sarcopenia, Nursing home

## Abstract

**Purpose:**

Sarcopenia is characterized by the loss of muscle mass, strength, and physical functioning. The bioelectrical impedance analysis (BIA) is a simplify method for the measurement of muscle quantity and quality. But there is a lack of evidence in the interpretation of the muscle quality parameter phase angle (PhA), which was recommended by the European Working Group on Sarcopenia in Older People 2 (EWGSOP2). We hypothesize that the PhA shows differences between sarcopenia categorized groups and can be used as an additional parameter for sarcopenia quantification among residents of nursing homes (NH).

**Methods:**

Based on EWGSOP2 specifications, 78 residents from five German NH was categorized into sarcopenia groups. Group comparisons with Kruskal–Wallis tests, Dunn–Bonferroni post-hoc-Tests, and correlations with Spearman coefficients were conducted with the muscle quality parameter PhA.

**Results:**

Significant group differences by Kruskal–Wallis test for PhA was detected (*H* = 8.150, *p* = 0.017). The Dunn–Bonferroni post-hoc-Test showed significant results by group comparison for “confirmed/ severe sarcopenia” (4.1° [3.1–5.0]) with “no sarcopenia” (4.6° [3.7–11.2]; *p* =0 .049) and “probable sarcopenia” (4.7° [3.4–13.5]; *p* = 0.016), respectively.

**Conclusions:**

There is a limitation for differentiation in preliminary stage of sarcopenia among multimorbid NH residents by PhA. Moreover, further research for specific cut-off-values and the individual sarcopenia progression monitoring by PhA are needed.

**Trial registration:**

No. AZ A2.5.4-096_aa (Date of approval: July 2019).

## Key summary points

**Aim:**

Can phase angle represents an additional parameter for quantifying sarcopenia among German nursing home residents?

**Findings:**

There is a limitation of the phase angle in differentiating between “confirmed/ severe sarcopenia” with “no sarcopenia” and “probable sarcopenia” groups among multimorbid nursing home residents in Germany.

**Message:**

Bioelectrical impedance analysis and determination of phase angle represent chances in the diagnostic of sarcopenia, but there is a limitation for differentiation in preliminary stage of sarcopenia among multimorbid NH residents.

## Introduction

Sarcopenia is a progressive musculoskeletal disease that increases the risk of falls, physical disability and mortality. Three categories are defined for the diagnosis of sarcopenia. The loss of muscle strength and muscle mass confirms sarcopenia. If there is also reduced physical functioning, severe sarcopenia is present [1, 2]. For the standardized quantification of sarcopenia, the European Working Group on Sarcopenia in Older People 2 (EWGSOP2) published recommendations with cut-off values and an algorithm for the European population in 2019 [3]. However, older people with increasing cognitive and physical limitations have difficulties in realizing the assessment methods. Limitations are particularly common among older persons in long-term care settings, such as nursing homes (NH). This could already be demonstrated in a pilot study of the larger BaSAlt project [4], which examined the feasibility of implementing the specified assessment methods of EWGSOP2 in German NH [5]. This circumstance shows that especially in settings with a high rate of multimorbid older persons, the quantification of sarcopenia should be simplified.

The bioelectrical impedance analysis (BIA) is an uncomplicated method for measuring the muscle mass. It is portable, inexpensive, and non-invasive in its application. The electrical resistance in the body is measured. In this way, various parameters of body composition can be detected [6]. To measure the bioelectrical impedance, a small alternating current is passed through the body. Two types of body resistance can be determined. The reactance (Xc) depends on the capacitance properties of the cell membranes. The measurement of Xc can thus be used to determine the body cell mass [7]. The resistance (R) depends on the total body water. It is the resistance of intra- and extracellular fluids or ionic solutions [8, 9]. For a prolonged period of time, body composition was determined using complex methods, such as dual x-ray analysis. For practical application in settings with vulnerable groups, BIA can be applied with comparable results [10–12]. The BIA in particular can demonstrate an opportunity for sarcopenia quantification in this setting due to its ease of use and evaluation [13].

The determination of the appendicular skeletal muscle mass (ASMM) with gender-specific cut-off values are recommended by the EWGSOP2 as a muscle quantity measurement [3, 14]. In their specification, EWGSOP2 also reports on additional measurement methods and tools for sarcopenia quantification. The measurement of muscle quality is a relatively new method for determining body composition and muscle functioning. One of these methods is the determination of the phase angle (PhA) [15], which is calculated (arctangent [Xc/R] × [180°/π]) and can be determined directly without population-specific factors. PhA describes changes in quality and quantity of soft tissue masses and can be an indicator of membrane integrity and intra- or extracellular water distribution [16, 17]. Moreover, it also serves as a predictor of body cell mass and nutritional status [18]. In general, PhA can be described as increasing over the life span into adulthood and reversible into old age [19].

However, there is no uniform consensus on the use of muscle quality parameters such as PhA in the context of sarcopenia by EWGSOP2 [3]. Further knowledge about the relationship between muscle quality and sarcopenia must follow in order to be able to give evidenced-based recommendations for the practical use. We hypothesize that PhA will provide an additional parameter for sarcopenia identification with differences in sarcopenia categorized groups by EWGSOP2 specification. This study examined the property of the PhA for sarcopenia quantification among German NH residents.

## Methods

### Recruitment, in-/exclusion criteria, and instruments

Fife NH in southwestern Germany have been included in the study. The first baseline survey (*t*1) took place between September 2020 and July 2021. A second recruiting for baseline survey (*t*2) was conducted in the already participating NH from August 2021 to April 2022. Before the measurement time t1, 11 assessors from the respective NH were trained for the assessments in a two-day workshop by the BaSAlt team.

Inclusion criteria for the study were a degree of care ≤ 4 (the German care system categorized the degree of care from 1 to 5). The voluntary participation in the study was a prerequisite. Exclusion criteria were a degree of care 5 and/ or a palliative condition with severe physical or mental disabilities. The Ethics Committee of the Faculty of Economics and Social Sciences of the Eberhard Karls University of Tübingen approved the project (no. AZ A2.5.4-096_aa).

Residents’ demographic/ anthropometric data sex, degree of care, age, height, weight, Body-Mass-Index (BMI), and the morbidity status were obtained from the medical file. The cognitive functioning was assessed using the Mini-Mental-Status-Test (MMST) [20]. The Barthel-Index (BI) was applied for the survey of the need of care [21]. The nutritional status of the residents was collected using the Mini-Nutrition-Assessment-Short-Form (MNA-SF©) [22]. Identify a possible risk of sarcopenia via a subjective self-assessment, the SARC-F questionnaire was used. A point score ≥ 4 points in total was interpreted as a predictor of sarcopenia [23]. According to EWGSOP2 guidelines, people with a SARC-F < 4 points are quantified as non-sarcopenic. However, the BaSAlt study uses an adapted algorithm, which indicates that the number of cognitive impaired residents is high and the validity of the self-assessment tool SARC-F in its assessment is not suitable. Therefore, SARC-F is not used as a quantification method in this study.

The muscle strength testing followed by hand force measurement. The maximum hand force (MHF) of the residents was tested using an isometric hand force dynamometer (Hydraulic Hand Force Dynamometer Saehan Model SH5001, Saehan, Changwon-si, Korea). Three measurements each were taken alternating the right and left hand. The best trial out of six was used for the maximum force value. The measurement was performed in sitting position and flexion (90°) in upper to lower arm. The cut-off values according to EWGSOP2 were < 16 kg for women and < 27 kg for men [24].

Confirmation of sarcopenia was checked by muscle mass determination. For this purpose, the measurement of appendicular skeletal muscle mass (ASMM) with BIA by Akern (Impedance Vector Analyzer BIA 101 BIVA, 50 kHz ± 1% measuring frequency) was performed. BIA measurement was taken in horizontal lying position without upper body inclination (0°) and abduction of arms (30°) and legs (45°), respectively. The measurement should be conducted in the morning before breakfast. Two electrodes were applied to the hand and foot segmentally of the right body half in a predetermined position and distance (> 5 cm) from each other. For further analysis, generated data was transferred into BodygramPlus Enterprise software (Version 1.2.2.9, Akern s.r.l., Pontassieve, Italy). ASMM cut-off values were < 15 kg for women and < 20 kg for men [25].

To determine the severity of sarcopenia, the physical functioning of the residents was assessed using the 4 m-walking-speed-Test. Habitual gait speed (4MWS) was recorded over a walking distance of four meters and measured using a stopwatch. Before and after the measured distance, run-on and run-off distances of two meters each are considered. The cut-off value for both sexes at the speed of ≤ 0.8 m/s was seated [26]. Residents who were not able to walk (e.g. wheelchair users) were classified as functional impaired.

**Fig. 1:**
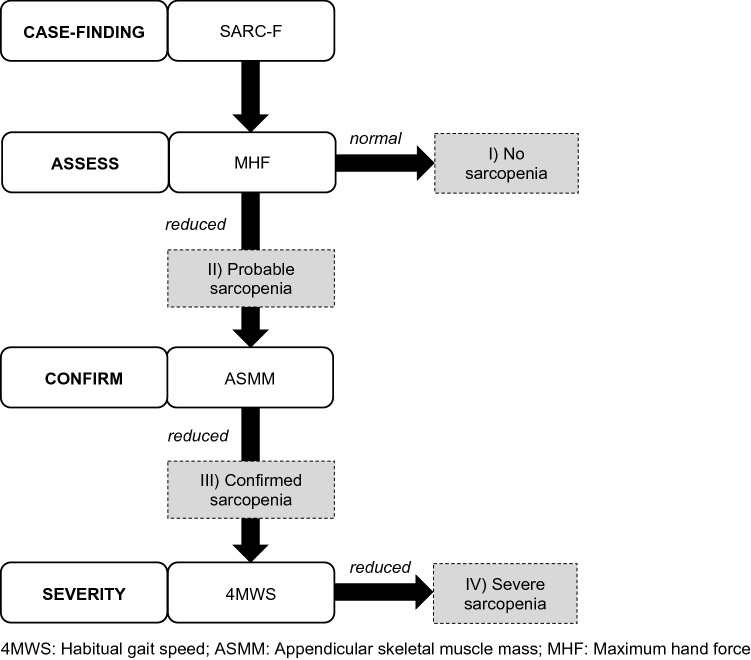
Sarcopenia quantification in the BaSAlt cohort-study (modified after [3])

The residents were categorized in four sarcopenia groups: (I) “no sarcopenia”, (II) “probable sarcopenia”, (III) “confirmed sarcopenia”, and (IV) “severe sarcopenia”. Figure 1 shows the sarcopenia quantification based on the adapted EWGSOP2 specifications in the BaSAlt cohort-study.

Further body composition parameters were recorded as a part of BIA measurement. The reactance (Xc) and resistance (Rz) were measured. Additionally, phase angle (PhA), fat mass (FM), fat-free mass (FFM), muscle mass (MM), total body water (TBW), extracellular water (ECW), and body cell mass (BCM) were calculated. For the parameters FM, FFM, and MM, the values adjusted for body size were also calculated.

## Data analysis

Data analysis was performed with the statistical program SPSS (IBM SPSS version 27.0.1.0). For descriptive analysis median values (Md) with range (minimum–maximum), mean with standard deviation, and percentages were collected. Normal distribution was calculated with Shapiro–Wilk–Test (*p*≥ .05). For the group comparisons, non-parametric analysis with Kruskal–Wallis-Tests were used. Post-hoc-Tests for comparison between the sarcopenia groups, adapted by Bonferroni-correction, were measured. The significance level was set at *p* ≤  0.05 for two-sided testing. Additionally, Pearson correlation coefficients was calculated for PhA and interpreted as small (|r| ≥  0.10), moderate (|*r*| ≥ 0.30), and large (|*r*| ≥ 0.50) effect by Cohen.

## Results

In total, 81 residents were included in the baseline survey for the measurement times *t*1 (*n* = 69) and *t*2 (*n* = 12). For three residents, an analysis of data could not be done, because one resident died, one resident left the NH, and another resident transitioned to palliative care during the measurement time t1.

For the sarcopenia quantification, the data from 78 residents from fife NH was analyzed. Missing values are described for the respective assessments and were not considered in the analysis. The evaluation of the morbidity status could be determined for 77 residents, because for one resident the access to the medical file was denied.

The quantification of sarcopenia in the BaSAlt cohort-study showed residents in the categories (I) “no sarcopenia” *n* = 32 (41.0%), (II) “probable sarcopenia” *n* = 33 (42.3%), (III) “confirmed sarcopenia” n = 1 (1.3%), and IV) “severe sarcopenia” *n* = 12 (14.4%). Due to the fact that only one resident was categorized into (III) “confirmed sarcopenia”, “confirmed/ severe sarcopenia” groups were summarized for further analysis. Assessed by the Shapiro–Wilk-Test, PhA was normally distributed for the group “confirmed/ severe sarcopenia” (*p* = 0.536). Non-normal distribution was seen for “no sarcopenia” and “probable sarcopenia” groups (both *p* < 0.001).

The evaluation of the MMST could not be determined for 12 residents. Reasons for this were blindness (*n* = 6), deafness (*n* = 1), lack of motivation (*n* = 4), and severe cognitive impairment (*n* = 1). The MNA-SF^®^ was not calculated for two residents, because the date of moving into the HN was to short (< 3 months). Due to a lack of motivation (*n* = 2) and severe cognitive impairment (*n* = 9), the evaluation of the SARC-F could not be determined for eleven residents. Also, MHF was not possible to assess for two residents, because they had severe cognitive impairment. The 4MWS were missed for 14 residents. Reasons for this were immobility without the help for walking in the NH from others (*n* = 4) or the use of a wheelchair (*n* = 10).

The study characteristic and sarcopenia quantification with Kruskal–Wallis-Tests, Dunn-Bonferroni post-hoc-Tests, and Pearson correlations between PhA and the other variables assessed in the BaSAlt cohort are shown in Table 1 and Table 2. In addition, BIA parameter for body composition and supplementary group descriptions are presented in Tables 3 and 4.

**Table 1:** Study characteristic of the BaSAlt cohort with group comparison for sarcopenia quantification, and Spearman correlation coefficients by PhA

	Degree of care Md (range) mean ± SD	Age (in years) Md (range) mean ± SD	Height (in cm) Md (range) mean ± SD	Weight (in kg) Md (range) mean ± SD	BMI (in kg/m^2^) Md (range) mean ± SD	Number of morbidities Md (range) mean ± SD	BI (in points) Md (range) mean ± SD	MMST (in points) Md (range) mean ± SD	MNA-SF^®^ (in points) Md (range) mean ± SD
No sarcopenia *n*_group_ = 32 *n*_female_ = 25 *n*_male_ = 7	3.0 (2–4) 2.94 ± 0.76	86.0 (65–98) 87.28 ± 7.20	162.5 (150.0–175.0) 172,75 ± 7.22	68.6 (41.4–101.0) 68.21 ± 11.22	25.0 (17.5–43.1) 25.81 ± 4.55	3.0 (1–5) 3.13 ± 1.16	80.0 (15–100) 73.28 ± 23.34	23.5 (4–30) 21.32 ± 7.45	12.0 (9–14) 12.10 ± 1,64
Probable sarcopenia *n*_group_ = 33 *n*_female_ = 21 *n*_male_ = 12	3.0 (2–4) 3.00 ± 0.61	87.0 (64–94) 85.67 ± 6.87	165.0 (144.0–187.0) 165.06 ± 9.02	73.1 (53.1–111.0) 75.72 ± 15.86	25.9 (20.9–45.4) 27.82 ± 5.65	3.0 (1–10) 3.53 ± 1.87	65.0 (10–100) 61.82 ± 22.74	19.0 (0–30) 19.26 ± 7.94	12.0 (8–14) 11.85 ± 156
Confirmed/ severe sarcopenia *n*_group_ = 13 *n*_female_ = 12 | *n*_male_ = 1	3.0 (2–4) 3.08 ± 0.64	89.0 (71–97) 89.15 ± 6.40	152.0 (140.0–174.0) 154.62 ± 10.20	57.4 (36.4–70.2) 54.96 ± 9.80	23.6 (15.5–28.9) 22.92 ± 3.42	2.0 (0–6) 2.85 ± 2.04	60.0 (25–75) 54.23 ± 16.69	15.0 (9–27) 17.73 ± 6.57	11.5 (3–13) 10.08 ± 3.15
Kruskal-Wallis-test	H = 0.427, *p* = *0.808*	H = 3.400, *p* = *0.183*	H = 10.899, *p* = 0.004*	H = 19.193, *p* < 0.001*	H = 9.681, *p* = 0.008*	H = 1.106, *p* = *0.575*	H = 10.919, *p* = 0.004*	H = 2.400, *p* = *0.301*	H = 4.795, *p* = *0.091*
Dunn–Bonferroni-post-hoc-tests									
No sarcopenia –probable sarcopenia	–	–	*z* = − 0.992, *p* = *0.964*	*z* = − 1.751, *p* = *0.240*	*z* = − 1.337, *p* = *0.543*	–	*z* = 2.337, *p* = *0.058*	–	–
No sarcopenia –confirmed, severe sarcopenia	–	–	z = 2.529, *p* = 0.034*	z = 3.039, *p* = 0.007*	z = 2.083, *p* = *0.112*	–	*z* = 1.296, *p* = *0.585*	–	–
Probable sarcopenia –confirmed, severe sarcopenia	–	–	*z* = 3.292, *p* = 0.003*	*z* = 4.379, *p* < 0.001*	*z* = 3.105, *p* = 0.006*	–	*z* = 3.053, *p* = 0.007*	–	–
Spearman correlation with PhA	r = 0.190, *p* = *0.095*	r = − 0.245, *p* = 0.031*	r = 0.088, *p* = *0.442*	r = − 0.089, *p* = *0.436*	r = − 0.048, *p* = *0.676*	r = − 0.084, *p* = *0.468*	r = − 0.080, *p* = *0.487*	r = − 0.140, *p* = *0.264*	r = − 0.015, *p* = *0.896*

*BI* Barthel-index, *BMI* body-mass-index, mini-mental-status-test, *MNA-SF*^*®*^ mini-nutrition-assessment-short-form

Essential is the indication of the *p*-values in italics, which are additionally marked as significant with a *

*Significant result for *p* ≤ 0.05 (two-sided test)

**Table 2:** Sarcopenia quantification with group comparison and Spearman correlation coefficients by PhA in the BaSAlt cohort

	SARC-F (in points) Md (range) mean ± SD	MHF (in kg) Md (range) mean ± SD	ASMM (in kg) Md (range) mean ± SD	4MWS (in m/s) Md (range) mean ± SD
No sarcopenia *n*_group_ = 32 *n*_female_ = 25, *n*_male_ = 7	3.0 (0–9) 3.17 ± 2.83	18.0 (16–40) 20.50 ± 5.99	16.3 (11.5–25.7) 17.37 ± 3.31	0.63 (0.27–0.95) 0.61 ± 0.19
Probable sarcopenia *n*_group_ = 33 *n*_female_ = 21, *n*_male_ = 12	3.0 (0–10) 3.36 ± 2.81	14.0 (2–26) 14.91 ± 6.44	19.7 (15.0–31.4) 20.11 ± 4.29	0.59 (0.32–1.02) 0.60 ± 0.18
Confirmed/ severe sarcopenia *n*_group_ = 13 *n*_female_ = 12, *n*_male_ = 1	4.5 (0–7) 4.10 ± 2.38	12.0 (0–26) 11.38 ± 6.12	13.2 (8.4–18.1) 12.97 ± 2.61	0.49 (0.28–0.90) 0.55 ± 0.18
Kruskal–Wallis-Test	*H* = 1.372, *p* = *0.504*	*H* = 25.991, *p* < 0.001*	*H* = 27.085, *p* < 0.001*	*H* = 0.966, *p* = *0.617*
Dunn–Bonferroni-post-hoc-tests				
No sarcopenia–probable sarcopenia	–	*z* = 3.672, *p* = 0.001*	*z* = − 2.484, *p* = 0.039*	–
No sarcopenia–confirmed/ severe sarcopenia	–	*z* = 1.943, *p* = *0.156*	*z* = 3.273, *p* = 0.003*	–
Probable sarcopenia–confirmed/ severe sarcopenia	–	*z* = 4.706, *p* < 0.001*	*z* = 5.170, *p* < 0.001*	–
Spearman correlation with PhA	*r* = − 0.106, *p* = *0.392*	r = 0.111, *p* = *0.338*	r = 0.477, *p* < 0.001*	r = − 0.001, *p* = *0.994*

Essential is the indication of the *p*-values in italics, which are additionally marked as significant with a *

*Significant result for *p* ≤ 0.05 (two-sided test)

**Table 3:** Body composition parameter with group comparison and Spearman correlation coefficients by PhA in the BaSAlt cohort

	Rz (in Ω/m) Md (range) mean ± SD	Xc (in Ω/m) Md (range) mean ± SD	PhA (in °) Md (range) mean ± SD	FM (in kg) Md (range) mean ± SD	FMI (in kg/m^2^) Md (range) mean ± SD	FFM (in kg) Md (range) mean ± SD	FFMI (in kg/m^2^) Md (range) mean ± SD	MM (in kg) Md (range) mean ± SD	MMI (in kg/m^2^) Md (range) mean ± SD	TBW (in l) Md (range) mean ± SD	ECW (in l) Md (range) mean ± SD	BCM(in kg) Md (range) mean ± SD
No sarcopenia *n*_group_ = 32 *n*_female_ = 25, *n*_male_ = 7	534.0 (367.8–680.7) 525.39 ± 75.31	42.0 (30.3–84.5) 44.53 ± 10.23	4.6 (3.7–11.2) 4.94 ± 1.46	20.8 (2.0–48.8) 19.48 ± 9.52	7.7 (0.7–20.8) 7.47 ± 3.91	46.7 (35.1–67.7) 48.74 ± 7.48	17.9 (14.8–22.3) 18.34 ± 1.97	18.6 (13.5–30.9) 20.40 ± 5.21	7.3 (5.3–10.4) 7.65 ± 1.54	34.5 (25.7–46.6) 36.14 ± 5.48	18.3 (13.4–24.5) 18.70 ± 2.78	21.3 (15.8–49.0) 23.19 ± 6.63
Probable sarcopenia *n*_group_ = 33 *n*_female_ = 21, *n*_male_ = 12	470.2 (217.3–714.2) 473.73 ± 98.70	41.1 (23.6–90.7) 42.18 ± 12.16	4.7 (3.4–13.5) 5.31 ± 2.17	19.5 (1.9–51.9) 22.42 ± 12.27	6.9 (0.8–22.5) 8.30 ± 4.84	53.1 (41.7–73.5) 53.27 ± 8.44	19.6 (14.8–24.0) 19.52 ± 2.36	24.0 (15.0–46.7) 24.60 ± 7.33	8.8 (5.1–18.2) 8.99 ± 2.46	39.7 (31.8–59.5) 40.95 ± 7.69	19.5 (11.4–36.5) 20.77 ± 5.11	24.6 (15.4–45.7) 25.88 ± 6.77
Confirmed/ severe sarcopenia *n*_group_ = 13 *n*_female_ = 12, *n*_male_ = 1	653.2 (482.2–803.2) 646.16 ± 95.50	43.2 (37.6–70.1) 46.52 ± 9.79	4.1 (3.1–5.0) 4.16 ± 0.55	17.2 (3.4–23.9) 15.78 ± 6.33	6.9 (1.5–10.4) 6.60 ± 2.63	39.3 (31.8–50.4) 39.16 ± 5.89	16.0 (14.1–20.2) 16.32 ± 1.62	13.8 (9.2–25.2) 14.33 ± 4.28	5.7 (4.2–8.5) 5.91 ± 1.24	29.1 (22.0–37.1) 28.67 ± 4.61	17.0 (11.5–19.5) 16.16 ± 2.38	16.8 (10.8–23.4) 16.59 ± 3.36
Kruskal-Wallis-Test	*H* = 2.201, *p* = *0.333*	*H* = 21.434, *p* < 0.001*	*H* = 8.150, *p* = 0.017*	*H* = 2.760, *p* = *0.252*	*H* = 0.895, *p* = *0.639*	*H* = 24.515, *p* < 0.001*	*H* = 17.885, *p* < 0.001*	H = 22.876, *p* < 0.001*	H = 21.829, *p* < 0.001*	H = 26.357, *p* < 0.001*	H = 11.862, *p* = 0.003*	H = 24.398, *p* < 0.001*
Dunn-Bonferroni-post-hoc-tests												
No sarcopenia–probable sarcopenia	–	*z* = 2.106, *p* = *0.106*	*z* = − 0.509, *p* = *0.999*	–	–	z = − 1.978, *p* = *0.144*	z = − 1.852, *p* = *0.192*	z = − 1.971, *p* = *0.146*	z = − 2.208, *p* = *0.082*	z = − 2.281, *p* = *0.068*	z = − 1.448, *p* = *0.443*	z = − 1.964, *p* = *0.148*
No sarcopenia–confirmed/ severe sarcopenia	–	*z* = − 3.002, *p* = 0.008*	*z* = 2.400, *p* = 0.049*	–	–	z = 3.435, *p* = 0.002*	z = 2.803, *p* = 0.015*	z = 3.271, *p* = 0.003*	z = 2.958, *p* = 0.009*	z = 3.375, *p* = 0.002*	z = 2.332, *p* = *0.059*	z = 3.434, *p* = 0.002*
Probable sarcopenia–confirmed/ severe sarcopenia	–	*z* = − 4.611, *p* < 0.001*	*z* = 2.796, *p* = 0.016*	–	–	z = 4.949, *p* < 0.001*	z = 4.218, *p* < 0.001*	z = 4.779, *p* < 0.001*	z = 4.644, *p* < 0.001*	z = 5.118, *p* < 0.001*	z = 3.440, *p* = 0.002*	z = 4.938, *p* < 0.001*
Spearman correlation with PhA	*r* = 0.434, *p* < 0.001*	*r* = − 0.482, *p* < 0.001*	–	*r* = − 0.257, *p* = 0.023*	r = − 0.265, *p* = 0.019*	r = 0.455, *p* < 0.001*	r = 0.564, *p* < 0.001*	r = 0.448, *p* < 0.001*	r = 0.487, *p* < 0.001*	r = 0.385, *p* < 0.001*	r = -0.223, *p* = *0.051*	r = 0.733, *p* < 0.001*

*BCM* body cell mass, *ECW* extracellular water, *FFM* fat-free mass, *FM* Fat mass, *MM* muscle mass, *PhA* phase angle, *TBW* total body water, *Rz* resistance, *Xc* reactance

Essential is the indication of the *p*-values in italics, which are additionally marked as significant with a *

*Significant result for *p* ≤ 0.05 (two-sided test)

**Table 4:** Study characteristic with classifications of the BaSAlt cohort

	No sarcopenia *n*_group_ = 32 *n*_female_ = 25 | *n*_male_ = 7	Probable sarcopenia *n*_group_ = 33 *n*_female_ = 21 | *n*_male_ = 12	Confirmed/ severe sarcopenia *n*_group_ = 13 *n*_female_ = 12 | *n*_male_ = 1
Degree of care–2	*n* = 10 (31.3%)	*n* = 6 (18.2%)	*n* = 2 (15.4%)
Degree of care–3	*n* = 14 (43.7%)	*n* = 21 (63.6%)	*n* = 8 (61.5%)
Degree of care–4	*n* = 8 (25.0%)	*n* = 6 (18.2%)	*n* = 3 (23.1%)
Past cardiovascular events	*n* = 9 (28.1%)	*n* = 10 (31.3%)	*n* = 4 (30.8%)
Arterial hypertension	*n* = 21 (65.6%)	*n* = 24 (75.0%)	*n* = 5 (38.5%)
Coronary heart disease	*n* = 4 (12.5%)	*n* = 10 (31.3%)	*n* = 3 (23.1%)
Cardiac insufficiency	*n* = 9 (28.1%)	*n* = 12 (37.5%)	*n* = 4 (30.8%)
Cardiac pacemaker	*n* = 3 (9.4%)	*n* = 3 (9.4%)	*n* = 1 (7.7%)
Post-stroke/cerebral hemorrhage/ TIA	*n* = 9 (28.1%)	*n* = 8 (25.0%)	*n* = 3 (23.1%)
Chronic lung disease	*n* = 1 (3.1%)	*n* = 5 (15.6%)	*n* = 1 (7.7%)
Cancer	*n* = 7 (21.9%)	*n* = 5 (15.6%)	*n* = 2 (15.4%)
Diabetes mellitus	*n* = 8 (25.0%)	*n* = 10 (31.3%)	*n* = 2 (15.4%)
Osteoarthritis of lower extremity	*n* = 8 (25.8%)	*n* = 9 (29.0%)	*n* = 2 (15.4%)
Psychological/ emotional/ nervous disease	*n* = 21 (65.6%)	*n* = 16 (50.0%)	*n* = 9 (69.2%)
BI–completely independent	*n* = 2 (6.3%)	*n* = 1 (3.0%)	*n* = 0 (0.0%)
BI–partially in need of care	*n* = 13 (40.6%)	*n* = 7 (21.2%)	*n* = 0 (0.0%)
BI–in need of care	*n* = 13 (40.6%)	*n* = 20 (60.6%)	*n* = 12 (92.3%)
BI–dependent on care	*n* = 4 (12.5%)	*n* = 5 (15.2%)	*n* = 1 (7.7%)
MMST–no dementia	*n* = 7 (25.0%)	*n* = 4 (14.8%)	*n* = 0 (0.0%)
MMST–mild cognitive impairment	*n* = 6 (21.4%)	*n* = 6 (22.2%)	*n* = 3 (27.3%)
MMST–mild dementia	*n* = 7 (25.0%)	*n* = 4 (14.8%)	*n* = 1 (9.1%)
MMST–moderate dementia	*n* = 7 (25.0%)	*n* = 11 (40.7%)	*n* = 6 (54.5%)
MMST–severe dementia	*n* = 1 (3.6%)	*n* = 2 (7.4%)	*n* = 1 (9.1%)
MNA-SF^®^–normal nutrition	*n* = 20 (64.5%)	*n* = 21 (63.6%)	*n* = 6 (50.0%)
MNA-SF^®^–risk of malnutrition	*n* = 11 (35.5%)	*n* = 12 (36.4%)	*n* = 4 (33.3%)
MNA-SF^®^–malnutrition	*n* = 0 (0.0%)	*n* = 0 (0.0%)	*n* = 2 (16.7%)
SARC-F–risk of sarcopenia	*n* = 11 (37.9%)	*n* = 9 (32.1%)	*n* = 9 (81.8%)
SARC-F–no risk of sarcopenia	*n* = 18 (62.1%)	*n* = 19 (67.9%)	*n* = 2 (18.2%)

*BI* Barthel-index, *MMST* mini-mental-status-test, *MNA-SF*^*®*^ mini-nutrition-assessment-short-form.

## Discussion

The BaSAlt cohort-study shows multimorbid NH residents in its results. Cognitive and physical impairments play key roles in sarcopenia quantification, as they severely limit the implementation of recommended assessment methods according to EWGSOP2 specification. Difficulties in implementation are mainly noticeable for the SARC-F and 4MWS. On the other hand, most residents were able to complete the required assessments for MHF and ASMM. This provides an opportunity for BIA to further sarcopenia diagnose using the muscle quality parameter PhA. But the specific focus on PhA and sarcopenia quantification has a lack of research that needs to be addressed by further studies.

Our study did not reveal any problems with the implementation of the BIA in the respective NH. The reason for this could be that it is used while the residents are measured in spine position. This leads to a higher acceptance among the residents and immobile residents can also be measured. Previous BIA measurements were implemented using BIA by standing position. However, it should be mentioned here that the comparison of different measurement methods and BIA devices is not recommended [27]. A standardization in the NH must take place in order to ensure a reliable data base. The limitation of comparability also refers to the cut-off values, which are given for the ASMM but not described for the PhA in the EWGSOP2 specifications. This must be enabled in prospective studies.

However, the differences within the age groups are not recorded more precisely, so that a rough orientation for the reference values is given. A systematic review by Di Vincenzo and colleagues (2021) examined PhA values specifically in relationship with sarcopenia. Significantly lower PhA values could be determined for sarcopenic individuals. Furthermore, higher prevalence of sarcopenia was recorded among people with lower PhA, such as cancer and geriatric patients [28]. Reference values for PhA were determined for the German population, but with a different BIA device and without setting-specific consideration than in our study [29]. From our point of view, these described references cannot be used for an evaluation of our BaSAlt cohort-study, as different sarcopenia quantification methods were collected in different settings and BIA devices. To the best of our knowledge, our study is the only one in Germany that has addressed the differences of PhA in relation to sarcopenia quantification among multimorbid NH residents according to EWGSOP2 specifications.

We hypothesized that PhA has the property for quantifying sarcopenia among NH residents. We can confirm this point, but we limit this statement due to the results of the comparisons of the three sarcopenia groups in our study. Accordingly, the comparisons of the groups showed significant differences only for the “no sarcopenia” and “probable sarcopenia” group compared to the “confirmed/ severe sarcopenia” group. It can be concluded that the prediction of PhA is limited in the preliminary stage of sarcopenia. Considering the prevention and treatment of sarcopenia, this seems to be an unfavorable outcome. Thus, especially in the transition from non-sarcopenic to pre-sarcopenic NH residents, no significance can be determined by the use of PhA. The study by Kołodziej et al. (2022) comes to a different conclusion. Accordingly, PhA is an additional parameter in the prognosis of pre-sarcopenia. Regular measurement using BIA is suggested among geriatric patients to minimize the risk of sarcopenia [30]. On the other hand, when considering sarcopenia, the individual is often forgotten due to various population-specific reference values and algorithms [31]. Measuring PhA over time span can also be used to document individual progress monitoring. Mainly for vulnerable individuals, such as NH residents, this could be a helpful tool to monitor the course and progression of the musculoskeletal disease sarcopenia.

For the standardized analysis of the PhA, trained staff, identical BIA devices, as well as time slots are needed. This should enable future reference values to provide scientific results. The BaSAlt team trained assessors in the use of the BIA device in standardized two training-days. In addition, the target for measuring body composition was set for the morning before breakfast in order to minimize shifts in fluid quantity and food intake. However, it was not always possible to satisfy that target, as the prevailing COVID-19 pandemic made it a priority to ensure primary care for NH residents. Furthermore, due to the pandemic situation and regulations for the uniform protection of residents in German long-term care settings, physical activity may have been negatively influenced. Physical activity shows positive correlations with increasing PhA [32]. Therefore, the contact restrictions and isolations of residents during the times of the COVID-19 pandemic may have influenced physical activity and thus PhA in our study.

Although, a limited number of residents were included in our study. This is reflected in the respective group sizes. Mainly the group of “confirmed sarcopenia” shows a low number of residents. We suspected a higher number of "confirmed sarcopenia" residents according to previous quantification studies. However, it is the implementation of assessment methods due to cognitive impairment and reduced physical functioning that shows difficulties. Mainly the SARC-F could not be answered by some residents and the validity remains questionable. The use of the PhA does not provide any additional benefit for sarcopenia quantification compared to previously used assay methods, such as MHF and ASMM. Due to the small sample size, PhA cannot be recommended as an additional parameter for sarcopenia quantification in our study.

## Conclusions

The use of PhA to quantify sarcopenia among NH residents remains questionable. The preliminary stage of sarcopenia cannot associate with the PhA. Further limitations are due to the lack of knowledge by PhA cut-off values for different BIA devices and the benefit for progression monitoring, which represent further goals in sarcopenia quantification according to EWGSOP2 specifications.

## Data Availability

On reasonable request, the datasets used and/or analyzed during the current study are available from the corresponding author.
